# A previously unreported impact of a *PLA2G7* gene polymorphism on the plasma levels of lipoprotein-associated phospholipase A2 activity and mass

**DOI:** 10.1038/srep37465

**Published:** 2016-12-01

**Authors:** Yue Qi, Dong Zhao, Zhangrong Jia, Wei Wang, Miao Wang, Jiayi Sun, Jun Liu, Yan Li, Wuxiang Xie, Jing Liu

**Affiliations:** 1 Beijing An Zhen Hospital, Capital Medical University; The Key Laboratory of Remodeling-Related Cardiovascular Diseases, Ministry of Education, Beijing Institute of Heart, Lung and Blood Vessel Diseases, Beijing, China

## Abstract

Lipoprotein-associated phospholipase A2 (Lp-PLA_2_) levels are associated with the development of atherosclerosis. We aimed to assess the genetic determinants of Lp-PLA_2_ activity and mass by genotyping multiple polymorphisms in *PLA2G7*, the gene encoding Lp-PLA_2_, among 1258 participants from the Chinese Multi-provincial Cohort Study-Beijing Project. The Sequenom MassARRAY system, Taqman assay and direct sequencing were adopted. For the first time, the rs13218408 polymorphism was found to be significantly associated with reduced Lp-PLA_2_ levels. We also confirmed the significant association of previously validated polymorphisms (rs1421378, rs1805018, rs16874954 and rs2216465), even after adjusting for traditional cardiovascular risk factors and for Bonferroni correction. Percentages of variance attributable to rs13218408 were 7.2% for activity and 13.3% for mass, and were secondary to those of rs16874954 (8.1% for activity and 16.9% for mass). A significant joint effect of rs13218408 and rs16874954 was observed on Lp-PLA_2_ activity (*P* = 0.058) and mass (*P* = 0.003), with their minor alleles together linking to the largest reduction in Lp-PLA_2_ levels (37.8% reduction in activity and 41.6% reduction in mass). Taken together, our findings show a significant association of a *PLA2G7* polymorphism with Lp-PLA_2_ levels, which was previously unreported in any population. The functionality of this genetic variation deserves further investigations.

Lipoprotein-associated phospholipase A2 (Lp-PLA_2_) is an enzyme produced by monocyte-macrophages, T-lymphocytes and other inflammatory cells^1^. In humans, Lp-PLA_2_ primarily circulates with low-density lipoproteins. It hydrolyses oxidised phosphatidylcholines and produces two proinflammatory molecules, namely lysophosphatidylcholine and oxidised free fatty acids^2^. Growing evidence supports a role of Lp-PLA_2_ in the pathogenesis of atherosclerosis^1^,^2^,^3^,^4^,^5^. Although several observational studies have suggested Lp-PLA_2_ activity or mass as an independent predictor for atherosclerotic cardiovascular disease (CVD)^5^ and the progression of subclinical atherosclerosis^6^,^7^,^8^,^9^, two recent large randomised, controlled, prospective clinical trials that selectively inhibited Lp-PLA_2_ failed to show benefits for clinical outcomes in patients with stable or unstable coronary artery diseases^10^,^11^. In light of conflicting findings from previous studies, exploring the determinants of Lp-PLA_2_ levels may be of clinical importance to identify those subjects with high Lp-PLA_2_ levels who will be more likely to experience a reduction in cardiovascular disease risk with Lp-PLA_2_ inhibition.

It is widely recognised that a strong genetic component underlies Lp-PLA_2_ activity and mass. Twin studies estimated the heritability of Lp-PLA_2_ activity and mass as 0.54 and 0.37, respectively^12^. A meta-analysis of genome-wide studies in 13,664 Caucasians revealed that genetic defects in *PLA2G7* (6p21.2-p12), the gene encoding Lp-PLA_2_, were significantly associated with plasma Lp-PLA_2_ activity and mass^13^. Studies on variants of the *PLA2G7* gene in general Asian populations demonstrated significant heterogeneity compared with Caucasians, and the frequency of alleles varied across different Asian groups^14^,^15^,^16^. Moreover, although multiple individual polymorphisms have been reported to be associated with Lp-PLA_2_ levels, whether there is a joint effect among these polymorphisms remains an open question. We therefore sought to explore *PLA2G7* polymorphisms associated with Lp-PLA_2_ activity and mass in a general Han Chinese population, and to test the joint effect of these polymorphisms on Lp-PLA_2_ activity and mass.

## Materials and Methods

### Study participants

Study participants were recruited from the Chinese Multi-provincial Cohort Study (CMCS)-Beijing Project, which is embedded in the CMCS, a nationwide population-based study investigating the risk factors related to the incidence of CVD^17^. In 1992, 1982 participants were enrolled for CMCS from a Beijing community using a stratified random sampling for each sex and a 10-year age group. From these, 1511 unrelated participants aged 45–74 years provided demographic characteristics and measurements of traditional risk factors from September to November in 2002. After excluding participants with established CVD (n = 73), hemolytic blood samples (n = 25), unavailable DNA samples (n = 107) and failed genotyping (n = 48), 1258 participants (592 male and 666 female) were analysed.

All participants provided informed consent. This study was reviewed and approved by the Ethics Committee of Beijing An Zhen Hospital, Capital Medical University, and was performed in accordance with standards set forth by the Declaration of Helsinki^18^.

### Risk factor survey

This study complied with the protocol set forth by the World Health Organization-MONICA (Monitoring of Trends and Determinants in Cardiovascular Disease). A standard questionnaire was designed to collect information on demographic characteristics, smoking status and personal medical history. Anthropometric measurements and blood pressure (BP) levels were recorded during physical examination. Body mass index (BMI) was calculated as weight in kilograms divided by height squared in metres. BP was measured in the right arm at a sitting position with a regular mercury sphygmomanometer after resting for at least 5 min. The mean value of two consecutive BP readings was used. Hypertension was defined as a mean systolic BP ≥ 140 mmHg and/or a mean diastolic BP ≥ 90 mmHg and/or currently on antihypertensive therapy^19^. Diabetes mellitus was defined as fasting blood glucose (FBG) ≥ 7.00 mmol/L and/or currently taking glucose-lowering medical treatments^20^. Regular smoking of one or more cigarettes per day was defined as current smoking.

### Laboratory assays

Venous blood samples were drawn from antecubital veins in the morning after fasting for at least 8 h. Fasting total cholesterol (TC), triglyceride (TG), low-density lipoprotein cholesterol (LDL-C), high-density lipoprotein cholesterol (HDL-C) and FBG were measured on the day of blood collection. TC, TG and FBG were determined by enzymatic methods; LDL-C and HDL-C were measured by homogeneous assays (Daiichi, Tokyo, Japan). The remaining samples were aliquoted and stored at −80 °C until used. Lp-PLA_2_ activity and mass were measured in 2012. A previous report confirmed that Lp-PLA_2_ activity and mass measurements in plasma-EDTA samples stored at −80 °C are stable after more than 10 years of storage^21^. Plasma Lp-PLA_2_ activity was measured using the Cayman colorimetric assay kit (Cayman Chemical Company, Ann Arbor, MI, USA). A pre-study validation was performed by analysing 20 samples for low-level control and 20 samples for high-level control in duplicate on consecutive days, and a Levey–Jennings chart was plotted^22^. The mean duplicate coefficient of variation (CV) was 7.31% for the low-level controls and 6.39% for the high-level controls. Plasma Lp-PLA_2_ mass was assayed according to the enzyme-linked immunoassay method using the diaDexus PLAC^®^ Test Kit (diaDexus, Inc., South San Francisco, CA, USA). The mean duplicate CV was 6.77% and 5.05% for low- and high-level controls, respectively. Because of limited plasma quantities, Lp-PLA_2_ mass measurements were conducted in 85% of samples (n = 1084) and run singly. To test whether this selection would lead to any potential bias affecting the validity of our findings, comparisons of major risk factors and levels of Lp-PLA_2_ activity were performed between samples for which Lp-PLA_2_ mass assays were and were not performed and no significant differences were observed (Table SI).

### Selection and genotyping of *PLA2G7* polymorphisms

**Figure 1:**
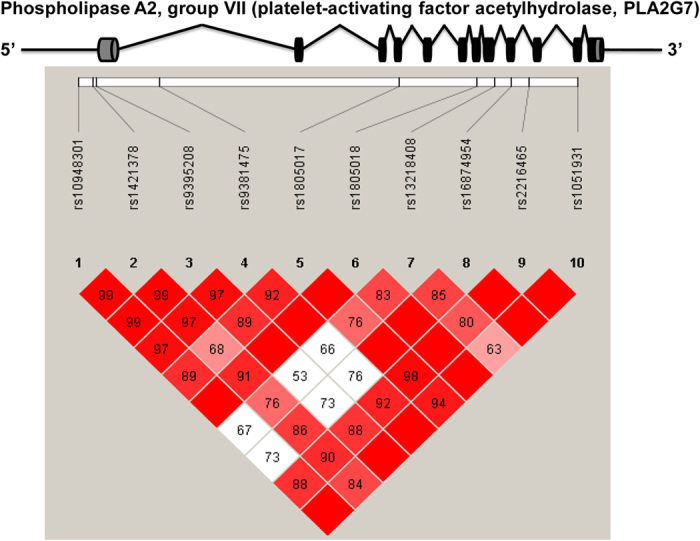
Pair-wise linkage disequilibrium among ten polymorphisms. The numbers inside the squares are D′ × 100.

Genomic DNA was extracted from white blood cells using the phenol/chloroform method and stored in 400 μl TE (10 mMTris-HCl, 1 mM EDTA, pH 8.0). Population-specific tagging polymorphisms with minor allele frequency (MAF) of at least 0.05 were selected from the HapMap PhaseII database using Haploview software (version 4.2) under the criteria r^2^ ≥ 0.8. Nine candidate polymorphisms were chosen and genotyped in this study according to a previously determined association between *PLA2G7* polymorphisms and Lp-PLA_2_ levels in studies of individual association, genome-wide studies and meta-analysis^23^,^24^,^25^, as well as whether a polymorphism is located in a functional region of *PLA2G7*. These included rs1805017 (R92H), rs1805018 (I198T), rs16874954 (V279F) and rs1051931 (A379V) in the *PLA2G7* coding region, rs10948301, rs1421378 and rs9395208 near the transcription start site, and rs9381475 and rs2216465 in *PLA2G7* introns. Genotyping was carried out on the Sequenom MassARRAY genotyping platform, and the call rates were more than 97% for the nine polymorphisms. The accuracy of our genotyping method was further confirmed by direct sequencing (BGI LifeTech, Beijing, China) of amplified DNA from 100 randomly selected samples and the discordance was less than 2% between the two methods. It is worth noting that during sequencing, we additionally detected a polymorphism, rs13218408, in intron 8 that was archived in the 1000GENOMES database on August 16, 2014. The association of this polymorphism with Lp-PLA_2_ levels had not been previously reported in any population; however, rs13218408 had strong association with plasma Lp-PLA_2_ activity and mass in the sequenced samples. Accordingly, rs13218408 was genotyped in whole samples by the Taqman genotyping assay kit (Applied Biosystems, Foster City, CA, USA) with call rates of 98%. Therefore, a total of ten selected polymorphisms were included and their pairwise linkage disequilibrium patterns are presented in Fig. 1.

### Study power estimation

In view of the partial determinant coefficients (R^2^) ranging from 0.007 to 0.04 between plasma Lp-PLA_2_ levels and examined polymorphisms, the estimated sample size in the present study was adequately powered (84.4%) such that the type I error probability (α) for a two sided-test was defined as 0.05, MAF was defined as 5% and partial R^2^ was estimated at 0.007.

### Statistical analysis

Continuous variables, expressed as the mean (standard deviation) for normal distributions or as medians (inter-quartile ranges), were compared by the unpaired Student’s t-test or the Mann–Whitney test between two groups and by one-way analysis of variance (ANOVA) across three or more groups. Categorical variables, expressed as numbers (percentages), were compared by the χ^2^ test. Spearman correlation coefficients were adopted to quantify the relationship between Lp-PLA_2_ activity and mass.

Deviation from Hardy–Weinberg equilibrium was tested by a Pearson goodness-of-fit test for all polymorphisms examined. After multiple comparisons, each polymorphism that showed significant association with Lp-PLA_2_ activity and mass was further adjusted for the known CVD risk factors, including age, sex, BMI, FBG, systolic BP, LDL-C, HDL-C and current smoking status by analysis of covariance. The percentage of variance of Lp-PLA_2_ activity and mass explained by each significant polymorphism was expressed as determinant coefficients in linear regression analyses. Considering linkage disequilibrium, five polymorphisms, rs1421378, rs1805017, rs13218408, rs16874954 and rs2216465, were selected in four respective haplotype blocks, and they were incorporated in a multiple linear regression model to evaluate their independent association with Lp-PLA_2_ levels. Interactive effects on Lp-PLA_2_ activity and mass between polymorphisms that are independently associated with Lp-PLA_2_ levels were further analysed by the general linear model.

Lipid-lowering medication has been reported to affect Lp-PLA_2_ levels. Therefore, the relationships of the examined polymorphisms with Lp-PLA_2_ activity and mass after adjusting for known CVD risk factors were also analysed with lipid-lowering treatment as a covariate in regression models or after excluding users of lipid-lowering medication.

The statistical analyses were computed with SPSS software (version 13.0; SPSS Inc, Chicago, IL, USA) and Haploview software (version 4.2; http://www.broad.mit.edu/mpg/haploview)^26^. Statistical power was calculated with Quanto software (version 1.2.3; http://hydra.usc.edu/gxe)^27^. All statistical tests were two-tailed, and *P* < 0.05 was considered statistically significant unless otherwise indicated.

## Results

### Characteristics of the study participants

Characteristics of the study participants are shown and compared between sexes in Table 1. Male participants had significantly higher plasma Lp-PLA_2_ activity and mass than female participants. There was a positive correlation between Lp-PLA_2_ activity and mass (Spearman correlation coefficient = 0.32, *P* < 0.001).

The MAF of rs13218408 was 8.1% in the present study, which was lower than that in East Asian samples recently described in the 1000GENOMES database. However, the MAFs of the other nine polymorphisms were similar to those in East Asian samples from the 1000GENOMES database (Table 2). For all polymorphisms examined, the genotype distributions were in Hardy–Weinberg equilibrium, and the genotype/allele distributions were comparable between sexes (Table SII).

### Effect of individual *PLA2G7* polymorphisms on Lp-PLA_2_ activity and mass

**Figure 2:**
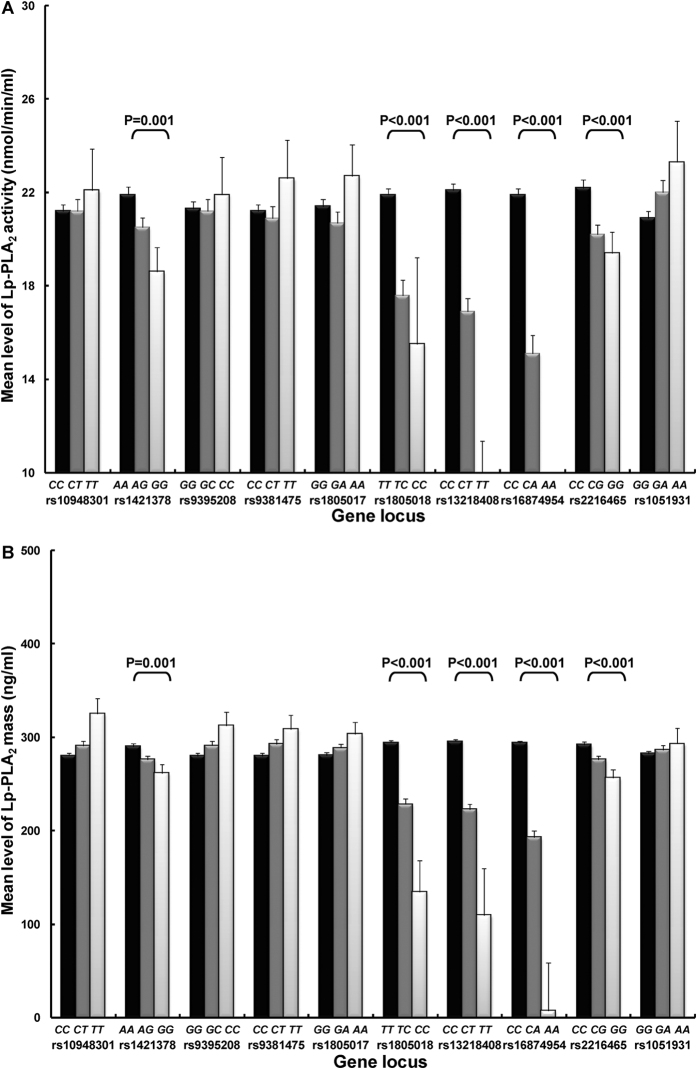
Levels of Lp-PLA_2_ activity (**A**) and mass (**B**) by genotypes of *PLA2G7* gene polymorphisms^†^. Data are presented as mean ± standard error. Differences were tested by the analysis of covariance (ANCOVA) after Bonferroni correction. ^†^Lp-PLA_2_ mass was measured in 1084 participants.

Five of the ten examined polymorphisms (rs1421378, rs1805018, rs13218408, rs16874954 and rs2216465) exhibited a significant association with Lp-PLA_2_ activity and mass after Bonferroni correction (*P* < 0.005) (Fig. 2). Carriers homozygous for the minor alleles of these five polymorphisms had the lowest levels of Lp-PLA_2_ activity and mass compared with the major allele homozygotes. The association of these polymorphisms with Lp-PLA_2_ activity and mass remained significant after adjustment for known CVD risk factors (Table 3). The proportion of variance of Lp-PLA_2_ activity and mass explained by each significant polymorphism ranged from 1.7% to 8.1% for activity, and from 1.4% to 16.9% for mass; the highest observed for rs16874954 (8.1% for activity and 16.9% for mass), followed by rs13218408 (7.2% for activity and 13.3% for mass). After excluding participants taking lipid-lowering medication or modelling lipid-lowering treatment as a covariate in regression analyses, there were still significant associations of these five polymorphisms with Lp-PLA_2_ activity and mass (data not shown).

Furthermore, to evaluate whether the association between the polymorphisms and Lp-PLA_2_ levels was independent of the other variants, a multiple linear regression analysis was conducted. Only rs13218408 and rs16874954 were independently associated with Lp-PLA_2_ activity and mass (Table SIII).

### Joint effect of *PLA2G7* polymorphisms on Lp-PLA_2_ activity and mass

**Figure 3:**
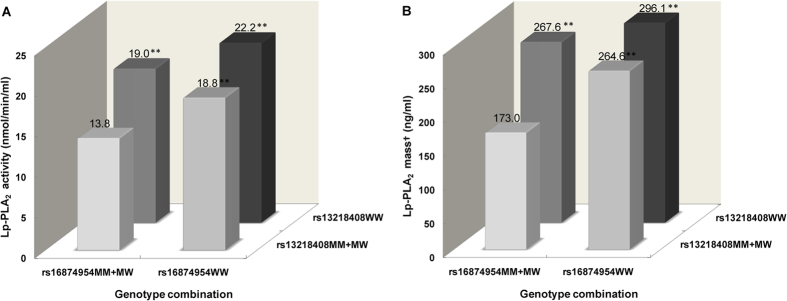
Joint effects of *PLA2G7* polymorphisms rs13218408 and rs16874954on Lp-PLA_2_ activity and mass. Abbreviations: Lp-PLA_2_, lipoprotein-associated phospholipase A_2_; MM, participants with minor allele homozygotes; MW, participants with heterozygotes; WW, participants with major allele homozygotes; BMI, body mass index; BP, blood pressure; FBG, fasting blood glucose; LDL-C, low-density lipoprotein cholesterol; HDL-C, high-density lipoprotein cholesterol. Data are expressed as mean ± standard deviation for continuous variables. Data were compared by analysis of covariance between subgroups with both the minor allele homozygotes and other subgroups. Association was adjusted for age, sex, BMI, systolic BP, FBG, LDL-C, HDL-C, and current smoking status. **P value < 0.001. ^†^Lp-PLA_2_ mass was measured in 1084 participants.

Genotype combination analysis was conducted for rs13218408 and rs16874954, the two polymorphisms that showed the strongest and independent association with Lp-PLA_2_ activity and mass (Table SIV). Interaction analysis showed that rs13218408 and rs16874954 had a significantly interactive effect on Lp-PLA_2_ mass (*P* = 0.003), and a marginally significant interactive effect on Lp-PLA_2_ activity (*P* = 0.058), as shown in Fig. 3. Participants who were simultaneous carriers for the minor alleles of these two polymorphisms (8.1% of study participants) had the lowest levels of Lp-PLA_2_ activity and mass compared with the other participants. This effect was independent of the known CVD risk factors.

## Discussion

The major finding of this study was that we found a significant association of the *PLA2G7* polymorphism, rs13218408, with the level of Lp-PLA_2_ activity and mass, which has not been reported previously in any population. Moreover, we also found a significant joint effect between this polymorphism and a widely validated coding polymorphism, rs16874954, on the level of Lp-PLA_2_.

Among the ten polymorphisms examined in this study, four were in the *PLA2G7* coding region, and two of them exhibited a marked association with Lp-PLA_2_ activity and mass in this population. Notably, polymorphism rs16874954 (V279F) is a widely evaluated locus in exon 9 with replacement of C by A, resulting in transversion of valine to phenylalanine. As indicated in our results, the heterozygous carriers of the rs16874954 minor allele had a significant reduction of 32.4% in Lp-PLA_2_ activity and 34.4% in mass, and almost no detectable enzyme activity and mass were found in the homozygous carriers. This loss of activity or mass caused by rs16874954 is supported by many previous reports^14^,^15^,^16^,^28^,^29^. Consistent with these observations, functional expression studies of the V279F mutation by Miwa *et al*.^28^ and Stafforini *et al*.^28^,^29^ demonstrated complete abolition of enzymatic activity and the molecular basis of an autosomal recessive form of Lp-PLA_2_ deficiency. Ishihara *et al*.^30^ and Zhang *et al*.^31^ reported that a complete absence of Lp-PLA_2_ activity in homozygous carriers of the rs16874954 minor allele was caused by a defect in enzyme secretion. Importantly, this polymorphism was mainly observed in Asians, with the highest frequency of the mutant allele found in Japanese (17.8%)^15^, and a lower frequency found in Koreans (12.6%)^14^ and Han Chinese (4.8% in this study, 5.4% in the study of Hou *et al*.^16^ and 6.5% in the study of Liu *et al*.^32^), whereas the polymorphism is rare in Europeans^13^. These findings highlight the genetic heterogeneity across ethnicities. In contrast to the Asian-specific nature of rs16874954 frequency, the other three non-synonymous *PLA2G7* polymorphisms, rs1805018 (I198T), rs1805017 (R92H) and rs1051931 (A379V), have been reported in multiple ethnic populations, and remarkable ethnic differences in the association with Lp-PLA_2_ activity and mass were found. For example, the minor allele 379 A of rs1051931 (A379V) was significantly associated with higher Lp-PLA_2_ activity in Caucasians^33^, but was not related to Lp-PLA_2_ levels in the present study or was significantly associated with lower Lp-PLA_2_ activity in Asians in a previous study^34^. Together, these findings suggest that the frequency and effect of *PLA2G7* coding polymorphisms on Lp-PLA_2_ levels might be influenced by divergent ethnic-specific genetic profiles.

Three non-coding polymorphisms were related to Lp-PLA_2_ levels in this study. It is notable that the polymorphism rs13218408 was found to be significantly associated with Lp-PLA_2_ levels for the first time. This polymorphism (in intron 8) has been identified in East Asian, African and European samples, with MAFs of 12.9%, 31.2% and 6.5%, respectively. A literature search did not reveal any evidence regarding its association with Lp-PLA_2_ levels or CVDs. Our study demonstrated a contributory role of rs13218408 to the variation of Lp-PLA_2_ activity and mass. This contribution (7.2% for activity and 13.3% for mass) was second only to rs16874954 (8.1% for activity and 16.9% for mass), calling for further validation in other populations. Further genetic combination analyses revealed significant interaction effects of rs13218408 and rs16874954 on Lp-PLA_2_ levels, and these effects were independent of traditional CVD risk factors. Although the linkage disequilibrium analyses showed that rs13218408 was in moderate linkage disequilibrium with rs16874954 and rs1805018, it is perhaps linked with one as-yet-unidentified functional polymorphism in or flanking *PLA2G7*. Another possible explanation for the biological relevance of rs13218408 is that it is located in a *PLA2G7* intron that may contain an enhancer element acting on *PLA2G7* or other genes in the vicinity, as supported by the HaploReg database. Despite all this, it is difficult to determine the underlying molecular mechanism causing these effects, and further studies are warranted.

The present study has several strengths. This is the first large-scale study conducted in a general Chinese population to investigate polymorphisms associated with two measures of Lp-PLA_2_ level (activity and mass). Moreover, for the first time, we identified a significant association of a *PLA2G7* polymorphism with Lp-PLA_2_ levels, and its combined influence with other *PLA2G7* polymorphisms on plasma levels of Lp-PLA_2_ activity and mass. Furthermore, to yield robust estimates, the known potential confounders that might affect Lp-PLA_2_ levels, such as CVD or lipid-lowering medications, were excluded or adjusted in a sensitive manner. Nevertheless, a number of potential limitations of this investigation merit careful consideration. First, we only focused on ten *PLA2G7* polymorphisms. These polymorphisms were chosen by a systematic review of the HapMap database and previous reports, and further strengthened by sequencing. However, we cannot exclude the impact of other unknown rare variants in the Chinese population. Second, the fact that our study participants were of Chinese descent may limit the generality of our findings, calling for further confirmation in other ethnic populations.

In summary, our findings identified a significant association of a *PLA2G7* polymorphism with the levels of Lp-PLA_2_, a potential risk factor of atherosclerotic CVD. Future studies that investigate the biological or clinical implications of this genetic variation are warranted.

## Additional Information

**How to cite this article**: Qi, Y. *et al*. A previously unreported impact of a *PLA2G7* gene polymorphism on the plasma levels of lipoprotein-associated phospholipase A2 activity and mass. *Sci. Rep.* **6**, 37465; doi: 10.1038/srep37465 (2016).

**Publisher's note:** Springer Nature remains neutral with regard to jurisdictional claims in published maps and institutional affiliations.

## Supplementary Material

Supplementary Information

## Figures and Tables

**Table 1:** Characteristics of the study participants.

Characteristic	Total	Male	Female	*P* value*
	n = 1258	n = 592	n = 666	
Age, years,	59.8 ± 7.9	61.4 ± 7.3	58.5 ± 8.1	<0.001
BMI, kg/m^2^	24.9 ± 3.3	24.9 ± 3.0	24.9 ± 3.5	0.935
Systolic BP, mmHg	129.6 ± 18.5	131.8 ± 18.2	127.7 ± 18.6	<0.001
Diastolic BP, mmHg	80.8 ± 10.1	83.1 ± 10.1	78.9 ± 9.7	<0.001
FBG, mmol/L	4.99 ± 1.22	5.03 ± 1.25	4.95 ± 1.20	0.271
Total cholesterol, mmol/L	5.57 ± 1.01	5.40 ± 0.97	5.73 ± 1.03	<0.001
LDL-C, mmol/L	3.36 ± 0.83	3.32 ± 0.81	3.41 ± 0.84	0.063
HDL-C, mmol/L	1.38 ± 0.31	1.29 ± 0.27	1.47 ± 0.31	<0.001
Triglycerides, mmol/L	1.35 (0.96–1.95)	1.34 (1.00–1.89)	1.37 (0.95–2.00)	0.886
Current smoking	121 (9.6)	118 (19.9)	3 (0.5)	<0.001
Hypertension	605 (48.1)	314 (53.0)	291 (43.7)	0.001
Diabetes	93 (7.4)	41 (6.9)	52 (7.8)	0.551
Hypertension treatment	373 (29.7)	183 (30.9)	190 (28.5)	0.356
Diabetes treatment	65 (5.2)	28 (4.7)	37 (5.6)	0.509
Lipid-lowering medication	140 (11.1)	60 (10.1)	80 (12.0)	0.291
Lp-PLA_2_ activity, nmol/min/ml	21.2 ± 8.3	22.8 ± 8.5	19.8 ± 7.7	<0.001
Lp-PLA_2_ mass, ng/ml†	283.7 ± 79.1	293.8 ± 80.2	274.7 ± 77.1	<0.001

Abbreviations: BMI, body mass index; BP, blood pressure; FBG, fasting blood glucose; LDL-C, low-density lipoprotein cholesterol; HDL-C, high-density lipoprotein cholesterol; Lp-PLA_2_, lipoprotein-associated phospholipase A_2_. Data are expressed as numbers (percentages) for categorical variables, as mean ± standard deviation for continuous variables in case of normal distributions and as medians (interquartile ranges) otherwise.

^*^P values for difference between males and females.

^†^Lp-PLA_2_ mass was measured in 1084 participants.

**Table 2:** Distribution of *PLA2G7* gene polymorphisms.

SNP	Locus	Minor Allele	Total, n (%)	Male, n (%)	Female, n (%)	MAF from 1000GENOMES database†				
						EUR	AFR	AMR	EAS	SAS
rs10948301	5′upstream	T	365 (14.5)	174 (14.7)	191 (14.3)	19.3%	11.3%	32.8%	17.2%	47.6%
rs1421378	5′upstream	G	582 (23.1)	250 (21.1)	332 (24.9)	39.6%	84.1%	49.3%	29.5%	68.3%
rs9395208	5′UTR	C	381 (15.1)	181 (15.3)	200 (15.0)	19.3%	11.3%	32.8%	17.4%	48.0%
rs9381475	Intron 1	T	375 (14.9)	178 (15.0)	197 (14.8)	21.2%	11.3%	33.3%	17.5%	46.9%
rs1805017	Exon 4 R92H	A	462 (18.4)	216 (18.2)	246 (18.5)	25.5%	25.7%	37.5%	21.9%	52.3%
rs1805018	Exon 7 I198T	C	199 (7.9)	74 (6.3)	125 (9.4)	5.5%	25.6%	3.2%	12.9%	12.5%
rs13218408	Intron 8	T	205 (8.1)	72 (6.1)	133 (10.0)	5.5%	29.9%	3.2%	12.9%	15.2%
rs16874954 (rs76863441)	Exon 9 V279F	A	121 (4.8)	48 (4.1)	73 (5.5)	0.0%	0.0%	0.00%	8.2%	0.0%
rs2216465	Intron 9	G	657 (26.1)	290 (24.5)	367 (27.6)	33.0%	42.1%	43.9%	35.0%	64.3%
rs1051931	Exon 11 V379A	A	372 (14.8)	180 (15.2)	192 (14.4)	24.3%	27.6%	15.4%	9.1%	14.4%

Abbreviations: MAF, minor allele frequency; EUR, European; AFR, African; AMR, Ad Mixed American; EAS, East Asian; SAS, South Asian.

^†^The MAF of polymorphisms was archived in the 1000GENOMES database on Aug 16, 2014.

**Table 3:** Levels of Lp-PLA_2_ activity and mass by genotypes of *PLA2G7* polymorphisms, after adjusting for cardiovascular risk factors.

SNP	Major-Allele Homozygotes			Heterozygotes			Minor-Allele Homozygotes			Percentage of Variance Explained	*P* value*
	No. of subjects	Geno-type	Mean level	No. of subjects	Geno-type	Mean level	No. of subjects	Geno-type	Mean level		
**Activity (nmol/min/ml)**											
rs1421378	749	AA	21.9 ± 8.3	436	AG	20.4 ± 8.2	73	GG	18.5 ± 7.7	1.7	<0.001
rs1805018	1065	TT	21.9 ± 8.2	187	TC	17.4 ± 7.3	6	CC	13.4 ± 16.6	4.2	<0.001
rs13218408	1060	CC	22.1 ± 8.1	191	CT	16.6 ± 7.1	7	TT	7.5 ± 7.1	7.2	<0.001
rs16874954	1140	CC	21.9 ± 8.1	115	CA	14.8 ± 6.5	3	AA	2.3 ± 0.8	8.1	<0.001
rs2216465	691	CC	22.1 ± 8.3	477	CG	20.3 ± 8.0	90	GG	19.1 ± 8.7	2.0	<0.001
**Mass (ng/ml)**†											
rs1421378	650	AA	290.2 ± 74.0	368	AG	276.1 ± 84.2	66	GG	261.5 ± 91.5	1.4	<0.001
rs1805018	916	TT	294.3 ± 74.4	163	TC	228.2 ± 75.6	5	CC	134.3 ± 128.5	10.4	<0.001
rs13218408	916	CC	295.6 ± 72.5	162	CT	222.6 ± 77.7	6	TT	109.9 ± 121.6	13.3	<0.001
rs16874954	978	CC	293.9 ± 73.8	104	CA	192.9 ± 58.3	2	AA	7.4 ± 0.6	16.9	<0.001
rs2216465	603	CC	292.1 ± 76.7	402	CG	276.3 ± 78.4	79	GG	256.6 ± 91.6	2.1	<0.001

Abbreviations: Lp-PLA_2_, lipoprotein-associated phospholipase A_2_; BMI, body mass index; BP, blood pressure; FBG, fasting blood glucose; LDL-C, low-density lipoprotein cholesterol; HDL-C, high-density lipoprotein cholesterol. Data are expressed as mean ± standard deviation.

^*^Adjusted for age, gender, BMI, systolic BP, FBG, LDL-C, HDL-C, and current smoking status by ANCOVA analysis.

^†^Lp-PLA_2_ mass was measured in 1084 participants.
