# Supplementary material for: A previously unreported impact of a PLA2G7 gene polymorphism on the plasma levels of lipoprotein-associated phospholipase A2 activity and mass
Source: Sci Rep. 2016 Dec 1;6:37465. doi: 10.1038/srep37465 (PMC5131362; doi:10.1038/srep37465)
Supplement: Supplementary Information [file srep37465-s1.pdf]

Supplementary Appendix

A previously unreported impact of a *PLA2G7* gene polymorphism on the plasma levels of lipoprotein-associated phospholipase A2 activity and mass

Yue Qi, Dong Zhao, Zhangrong Jia, Wei Wang, Miao Wang, Jiayi Sun, Jun Liu, Yan Li, Wuxiang Xie, Jing Liu*

Authors' affiliation:

Beijing An Zhen Hospital, Capital Medical University; The Key Laboratory of Remodeling-Related Cardiovascular Diseases, Ministry of Education; Beijing Institute of Heart, Lung and Blood Vessel Diseases, Beijing, China.

* Correspondence should be addressed to Jing Liu (jingliu@ccmu.edu.cn)

Index

Supplementary Tables (1-4) 2-7

Supplementary Tables

Supplementary Table SI. Characteristics among participants tested and untested for Lp-PLA_2 mass levels[†]

Characteristic	Participants who tested for mass n=1084	Participants who untested for mass n=174	*P* value*
Age, years,	59.8 ± 7.8	60.2 ± 8.2	0.483
Male	509 (47.0)	83 (47.7)	0.855
BMI, kg/m^2	24.9 ± 3.3	24.8 ± 3.3	0.711
Systolic BP, mmHg	129.4 ± 18.8	131.4 ± 16.8	0.186
Diastolic BP, mmHg	80.8 ± 10.3	81.0 ± 8.8	0.762
FBG, mmol/L	4.97 ± 1.19	5.09 ± 1.44	0.229
Total cholesterol, mmol/L	5.56 ± 1.02	5.64 ± 1.00	0.329
LDL-C, mmol/L	3.35 ± 0.83	3.43 ± 0.81	0.293
HDL-C, mmol/L	1.38 ± 0.30	1.42 ± 0.32	0.060
Triglycerides, mmol/L	1.34 (0.97–1.93)	1.43 (0.90–1.99)	0.876
Current smoking status	103 (9.5)	18 (10.3)	0.726
Hypertension	519 (47.9)	86 (49.4)	0.705
Diabetes	82 (7.6)	11 (6.3)	0.561
Hypertension treatment	318 (29.3)	55 (31.6)	0.542
Diabetes treatment	56 (5.2)	9 (5.2)	0.997
Lipid-lowering medication	126 (11.6)	14 (8.0)	0.164

Lp-PLA$_2$ activity, nmol/min/ml	21.2 ± 8.2	21.0 ± 8.3	0.714

Abbreviations: Lp-PLA$_2$, lipoprotein-associated phospholipase A$_2$; BMI, body mass index; BP, blood pressure; FBG, fasting blood glucose; LDL-C, low-density lipoprotein cholesterol; HDL-C, high-density lipoprotein cholesterol.

Data are expressed as numbers (percentages) for categorical variables, as mean ± standard deviation for continuous variables in case of normal distributions and as medians (interquartile ranges) otherwise.

† Lp-PLA$_2$ mass was measured in 1084 participants.

***** *P* values were calculated between participants tested and untested for Lp-PLA$_2$ mass levels.

Supplementary Table SII. Distribution of *PLA2G7* gene polymorphisms

SNP	Locus	Genotype / Allele	Total n=1258 n (%)	Male n=592 n (%)	Female n=666 n (%)	HWE *P* value for all
rs10948301	5'upstream	CC	917 (72.9)	428 (72.3)	489 (73.4)	
		CT	317 (25.2)	154 (26.0)	163 (24.5)	0.854
		TT	24 (1.9)	10 (1.7)	14 (2.1)	
		T	365 (14.5)	174 (14.7)	191 (14.3)	
rs1421378	5'upstream	AA	749 (59.5)	373 (63.0)	376 (56.5)	
		AG	436 (34.7)	188 (31.8)	248 (37.2)	0.666
		GG	73 (5.8)	31 (5.2)	42 (6.3)	
		G	582 (23.1)	250 (21.1)	332 (24.9)	
rs9395208	5'UTR	GG	906 (72.0)	422 (71.3)	484 (72.7)	
		GC	323 (25.7)	159 (26.9)	164 (24.6)	0.999
		CC	29 (2.3)	11 (1.8)	18 (2.7)	
		C	381 (15.1)	181 (15.3)	200 (15.0)	
rs9381475	Intron 1	CC	910 (72.3)	424 (71.6)	486 (73.0)	
		CT	321 (25.5)	158 (26.7)	163 (24.5)	0.978
		TT	27 (2.2)	10 (1.7)	17 (2.5)	
		T	375 (14.9)	178 (15.0)	197 (14.8)	
rs1805017	Exon 4	GG	841 (66.8)	395 (66.7)	446 (67.0)	
	Arg92His	GA	372 (29.6)	178 (30.1)	194 (29.1)	0.889
		AA	45 (3.6)	19 (3.2)	26 (3.9)	

		A	462 (18.4)	216 (18.2)	246 (18.5)	
rs1805018	Exon 7	TT	1065 (84.6)	520 (87.9)	545 (81.8)	
	Ile198Thr	TC	187 (14.9)	70 (11.8)	117 (17.6)	0.770
		CC	6 (0.5)	2 (0.3)	4 (0.6)	
		C	199 (7.9)	74 (6.3)	125 (9.4)	
rs13218408	Intron 8	CC	1060 (84.3)	522 (88.2)	538 (80.8)	
		CT	191 (15.2)	68 (11.5)	123 (18.5)	0.878
		TT	7 (0.5)	2 (0.3)	5 (0.7)	
		T	205 (8.1)	72 (6.1)	133 (10.0)	
rs16874954	Exon 9	CC	1140 (90.6)	545 (92.0)	595 (89.3)	
(rs76863441)	Val279Phe	CA	115 (9.2)	46 (7.8)	69 (10.4)	0.998
		AA	3 (0.2)	1 (0.2)	2 (0.3)	
		A	121 (4.8)	48 (4.0)	73 (5.5)	
rs2216465	Intron 9	CC	691 (54.9)	344 (58.1)	347 (52.1)	
		CG	477 (37.9)	206 (34.8)	271 (40.7)	0.827
		GG	90 (7.2)	42 (7.1)	48 (7.2)	
		G	657 (26.1)	290 (24.5)	367 (27.5)	
rs1051931	Exon 11	GG	911 (72.4)	424 (71.6)	487 (73.1)	
	Val379Ala	GA	322 (25.6)	156 (26.4)	166 (24.9)	0.855
		AA	25 (2.0)	12 (2.0)	13 (2.0)	
		A	372 (14.8)	180 (15.2)	192 (14.4)	

Data were presented as numbers (percentages).

Supplementary Table SIII. Multiple linear regression analysis of factors associated with Lp-PLA_2 activity and mass

Variable	Coefficient	SE	t	*P* value*
**Activity**				
Male	1.770	0.469	3.776	<0.001
Systolic BP, mm/Hg	0.042	0.012	3.458	0.001
LDL-C, mmol/L	3.014	0.272	11.095	<0.001
HDL-C, mmol/L	-4.949	0.778	-6.360	<0.001
rs13218408 CT+TT	-3.778	0.859	-5.890	<0.001
rs16874954 CA+AA	-5.590	0.993	-5.631	<0.001
**Mass**†				
Age, years	1.233	0.294	4.200	<0.001
Male	23.981	4.493	5.337	<0.001
LDL-C, mmol/L	5.558	2.572	2.161	0.031
HDL-C, mmol/L	61.034	7.352	8.302	<0.001
rs13218408 CT+TT	-42.112	8.127	-5.182	<0.001
rs16874954 CA+AA	-82.063	9.390	-8.740	<0.001

Abbreviations: Lp-PLA_2, lipoprotein-associated phospholipase A_2; BMI, body mass index; BP, blood pressure; FBG, fasting blood glucose; LDL-C, low-density lipoprotein cholesterol; HDL-C, high-density lipoprotein cholesterol.

† Lp-PLA_2 mass was measured in 1084 participants.

* BMI, FBG, current smoking status, rs1421378, rs1805017, and rs2216465 were also considered in the multiple linear regression model, but they were non-significant and not included in the model.

Supplementary Table SIV. Joint effects of *PLA2G7* polymorphisms rs16874954 and rs13218408 on Lp-PLA_2 activity and mass

Genotype combination*	Number (percent)	Activity (nmol/min/ml)	Mass† (ng/ml)
rs16874954 & rs13218408			
rs16874954 (-) – rs13218408 (-)	1044 (83.0)	22.2 ± 8.1	296.1 ± 72.6
rs16874954 (-) – rs13218408 (+)	16 (1.3)	18.8 ± 9.1[a]	264.6 ± 59.6[a]
rs16874954 (+) – rs13218408 (-)	96 (7.6)	19.0 ± 7.6[b]	267.6 ± 81.9[a]
rs16874954 (+) – rs13218408 (+)	102 (8.1)	13.8 ± 6.0[b]	173.0 ± 60.5[b]

Abbreviations: Lp-PLA_2, lipoprotein-associated phospholipase A_2; BMI, body mass index; BP, blood pressure; FBG, fasting blood glucose; LDL-C, low-density lipoprotein cholesterol; HDL-C, high-density lipoprotein cholesterol.

Data are expressed as numbers (percentages) for categorical variables, as mean ± standard deviation for continuous variables.

* (-) denotes major-allele homozygotes; (+) denotes minor-allele carriers.

† Lp-PLA_2 mass was measured in 1084 participants.

Data were compared by analysis of covariance between subgroups of both major-allele homozygotes and other subgroups after adjusting for age, sex, BMI, systolic BP, FBG, LDL-C, HDL-C, and current smoking status.

[a] P value < 0.01; [b] P value < 0.001.
